# Nanomedicine Approaches for the Pulmonary Treatment of Cystic Fibrosis

**DOI:** 10.3389/fbioe.2019.00406

**Published:** 2019-12-17

**Authors:** Cecilia Velino, Francesca Carella, Alessio Adamiano, Maurizio Sanguinetti, Alberto Vitali, Daniele Catalucci, Francesca Bugli, Michele Iafisco

**Affiliations:** ^1^Institute of Science and Technology for Ceramics (ISTEC), National Research Council (CNR), Faenza, Italy; ^2^Fondazione Policlinico Universitario “A. Gemelli” IRCCS, Dipartimento di Scienze di Laboratorio e Infettivologiche, Rome, Italy; ^3^Istituto di Microbiologia, Università Cattolica del Sacro Cuore, Rome, Italy; ^4^Institute for the Chemistry of Molecular Recognition (ICRM), National Research Council (CNR), c/o Institute of Biochemistry and Clinical Biochemistry, Catholic University, Rome, Italy; ^5^Humanitas Clinical and Research Center, Rozzano, Italy; ^6^Institute of Genetic and Biomedical Research (IRGB) - UOS Milan, National Research Council (CNR), Milan, Italy

**Keywords:** cystic fibrosis, nanoparticles, drug delivery, gene therapy, lung pathology

## Abstract

Cystic fibrosis (CF) is a genetic disease affecting today nearly 70,000 patients worldwide and characterized by a hypersecretion of thick mucus difficult to clear arising from the defective CFTR protein. The over-production of the mucus secreted in the lungs, along with its altered composition and consistency, results in airway obstruction that makes the lungs susceptible to recurrent and persistent bacterial infections and endobronchial chronic inflammation, which are considered the primary cause of bronchiectasis, respiratory failure, and consequent death of patients. Despite the difficulty of treating the continuous infections caused by pathogens in CF patients, various strategies focused on the symptomatic therapy have been developed during the last few decades, showing significant positive impact on prognosis. Moreover, nowadays, the discovery of CFTR modulators as well as the development of gene therapy have provided new opportunity to treat CF. However, the lack of effective methods for delivery and especially targeted delivery of therapeutics specifically to lung tissues and cells limits the efficiency of the treatments. Nanomedicine represents an extraordinary opportunity for the improvement of current therapies and for the development of innovative treatment options for CF previously considered hard or impossible to treat. Due to the peculiar environment in which the therapies have to operate characterized by several biological barriers (pulmonary tract, mucus, epithelia, bacterial biofilm) the use of nanotechnologies to improve and enhance drug delivery or gene therapies is an extremely promising way to be pursued. The aim of this review is to revise the currently used treatments and to outline the most recent progresses about the use of nanotechnology for the management of CF.

## Introduction

**Figure 1:**
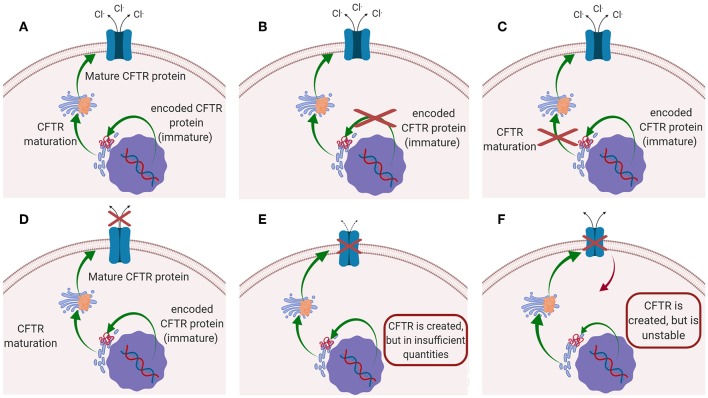
Schematic representation of the mutations on CFTR leading to CF disease: **(A)** CFTR works normally (no mutations); **(B)** Class I and VII mutation; **(C)** Class II mutations; **(D)** classes III and IV mutations; **(E)** Class V mutation; **(F)** class VI mutations.

Cystic fibrosis (CF) is the most common autosomal recessive disease, affecting today nearly 70,000 patients worldwide (Savla and Minko, 2013; da Silva et al., 2017; Haque et al., 2018), while ~1,000 new cases are diagnosed every year (Cystic Fibrosis Foundation, 2018). The disease is caused by mutations in the gene located on chromosome 7, that encodes the cystic fibrosis transmembrane conductance regulator (CFTR) protein. CFTR protein works as a chloride channel on the apical membrane of epithelial cells (Turcios, 2005; Høiby, 2011; Robinson et al., 2018), and it is responsible for the regulation of chloride ions secretion and sodium ions reabsorption (Ibrahim et al., 2011b). Since the identification of CFTR gene in 1989, more than 1,500 mutations have been described, each leading to CF disease (Ratjen, 2009). Those mutations have been initially classified in five major classes according to their effect on CFTR function (Figure 1): (i) mutations interfering with protein synthesis, (ii) mutations affecting protein maturation, (iii) mutations altering channel regulation, (iv) mutations affecting chloride conductance, and (v) mutations reducing the level of normally functioning CFTR at the apical membrane (Fanen et al., 2014). This classification system has been updated by De Boeck and Amaral (2016), which introduced two more classes: class (vi) includes mutations which destabilize CFTR at the cell surface, while mutations of class (vii), called “unrescuable” have the same outcomes as class (i) (i.e., the absence of CFTR protein), but they cannot be rescued by corrective therapy (Amaral, 2015; De Boeck and Amaral, 2016) (Figure 1). Among all the mutations, the most common is named F508del, which, through the deletion of phenylalanine at position 508 of the CFTR protein (O'Sullivan and Freedman, 2009; Gaspar et al., 2013; McNeer et al., 2015), prevents the protein from trafficking to the cell surface, thus significantly reducing CFTR function. It was estimated that 90% of CF patients carry at least one copy of this mutated form (Cartiera et al., 2010).

The consequence of mutations on CFTR results in impaired chloride ions secretion and hyper-absorption of sodium ions across epithelia (Cristallini et al., 2019), causing a hypersecretion of thick mucus difficult to clear. The over-production of the mucus secreted in the lungs, along with its altered composition and consistency, results in airway obstruction that makes the lungs susceptible to recurrent and persistent bacterial infections (Bahadori and Mohammadi, 2012) and endobronchial chronic inflammation, which are considered the primary cause of bronchiectasis, respiratory failure, and consequent death of patients (Henke and Ratjen, 2007). The presence of abnormal thick and viscous mucus impairs the mucociliary clearance and favors the bacterial colonization. The microorganisms residing inside the immobile mucus find a perfect environment to settle and are shielded against immune response as well as against antimicrobial drugs. Although the most severe problems related to CF, in terms of symptoms and treatments, involve the lungs (Davies et al., 2007), patients affected by CF often suffer of several other diseases occurring within other epithelial-lined organs such as small intestine bacterial overgrowth, pancreatic exocrine insufficiency, cirrhosis of the liver and progressive hepatic dysfunction, and infertility (Ramsey, 1996).

**Figure 2:**
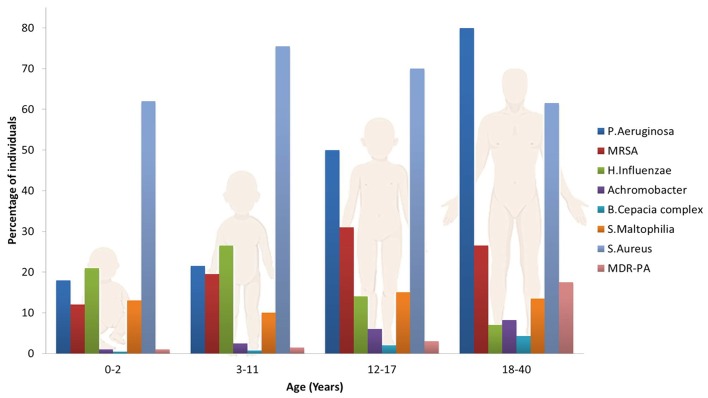
Prevalence of microorganisms in the lungs of CF patients as a function of age.

The most common pathogen affecting the airways in CF patients is *Pseudomonas Aeruginosa* (PA), although other microorganisms may play an important role on the pathogenesis of lung function declines, such as *Haemophilius influenzae, Staphilococcus aureus, Stenotrophomonas maltophilia, Burkholderia cepacian* (Rowe et al., 2005; Döring et al., 2012; d'Angelo et al., 2014) and the filamentous fungus *Aspergillus fumigatus* which often co-colonizes the lungs with PA (Zhao et al., 2018). This set of pathogens are frequently acquired in an age dependent sequence (Figure 2): *S. Aureus* and *H. Influenzae* are the most prevalent bacterial pathogens in younger patients, while PA chronically infects 80% of CF patients by late adolescence. Other pathogens, such as *B. Cepacia* and *S. Maltophilia* are less frequent but particularly troublesome in CF patients due to their multi-drug resistant phenotypes. The frequency of infections caused by these species increases with patient age, resulting in a significant health risk to CF patients surviving to adulthood (Pompilio et al., 2018). The U.S. patent registry reported that in 2017 the median age at death was 30.7 years, while about 15% of deaths occurred before 20 years of age (Cystic Fibrosis Foundation, 2018).

PA belongs to the family of Gram-negative organisms (Alikhani et al., 2018). Due to its wide genetic diversity, it can persist in CF patients' lungs and, if established, remains very difficult to eradicate. The initial infection usually involves a “non-mucoid” strain of the pathogen that can be cleared by the host or eradicated with an aggressive antibiotic treatment. Over time, as the colonization advances, PA colonies alter their pattern of gene expression in the CF lung and start producing a mucoid coating made of alginate which protects the bacteria against antibiotics and phagocytosis (Turcios, 2005; O'Sullivan and Freedman, 2009; Gaspar et al., 2013; Bhagirath et al., 2016). In this mucoid form, PA is able to form a biofilm which makes the pathogen up to 100 times more tolerant to antimicrobial treatments (Al-Obaidi et al., 2018; Ernst et al., 2018; Kłodzinska et al., 2018; Lu et al., 2018). Besides, the ability of PA to establish drug resistant biofilms is thought to be facilitated by the hypersecretion of the thick and viscous mucus layer in the CF airway, which provides a low oxygen environment (Moreau-Marquis et al., 2008). Essentially, both the presence of thick bronchiolar mucus and bacterial biofilm contribute to poor lung penetration of antimicrobial agents, thus leading to clinical aggravation and inefficacy of the therapies.

## Current Therapies

Despite the difficulty of treating the continuous infections caused by PA and other pathogens in CF patients, various treatment strategies have been developed during the last few decades, showing significant positive impact on prognosis (Heijerman et al., 2009).

Together with other organs, such as skin and intestines, the lungs are in direct contact with the external environment (Yang et al., 2008). Systemic delivery of drugs via inhalation routes (oral and nasal) constitutes an effective alternative to parenteral drug delivery to treat pulmonary disease. There are several advantages in drug administration via pulmonary route, including rapid onset of action, non-invasive nature, high permeability of the lungs (Sung et al., 2007), rapid absorption (Paranjpe and Müller-Goymann, 2014) and improved patient compliance. Hence, this strategy has been used for local delivery of several types of drugs, from antibiotics to chemotherapeutics, from peptides to vaccines (Muralidharan et al., 2015). Nowadays all the most used antimicrobials for CF such as tobramycin, colistimethate sodium, and aztreonam lysine (Littlewood et al., 2012) have been developed as inhalation formulations, as reported below in more details.

For long time the research for CF treatment was mainly focused on the symptomatic therapy, aimed at attenuating disease progression and delaying the onset of lung damage through different types of drugs. However, most of these therapies only treat the symptoms of CF, without correct the underlying causes of the disease (Savla and Minko, 2013). Nowadays, the discovery of CFTR modulators that aim to increase or potentially restore the function of the disease-causing CFTR protein (Lopes-Pacheco, 2016) as well as the development of gene therapy have provided new opportunities to treat CF (Perry et al., 2017).

### Symptomatic Therapy

Currently, antibiotics and anti-inflammatory drugs are widely used for the symptomatic treatment of CF related infections and inflammations. In addition, bronchodilators, mucolytics and osmotic agents are administered to improve airway and sputum clearance. As the airways are a major therapeutic target in the treatment of CF, many drugs are delivered by inhalation by using nebulizers, pressurized metered dose inhalers (pMDIs) and dry powder inhalers (DPIs). Nebulizers were the first devices that appeared in the market followed by pMDIs and DPIs. Characteristics of these devices are reported in Table 1. The main benefit of topical inhalation is the delivery of relatively high doses of the drug directly to the target site, while minimizing the risk of ototoxicity and nephrotoxicity (Abramowsky and Swinehart, 1982; Ramsey, 1996; Geller, 2009; Agent and Parrott, 2015).

**Table 1:** Main characteristics of the devices currently used for pulmonary delivery.

**Device**	**Mechanism**	**Characteristics**	**Disadvantages**
Nebulizer	Nebulization by air-jet	Vibrating mesh technology Aerosol droplets generated from liquids	Long inhalation times Cleaning times Frequent administration
Pressurized metered dose inhaler (pMDI)	Use of propellant	Aerosol droplets generated from a drug suspension in volatile liquid Inexpensive Correct size of particles deposited in the lungs	Lung deposition efficacy <60% Propellant requirement
Dry powder inhaler (DPI)	Dry powder	High stability and sterility Small portable devices Short administration	High inspiratory effort to be efficient

#### Antibiotics

##### Tobramicyn

Tobramicyn is an aminoglycoside antibiotic that has been widely used to treat various types of bacterial infections, especially those caused by gram-negative bacteria, such as PA (Geller et al., 2003; Van Westreenen and Tiddens, 2010). Tobramycin acts as a bactericidal drug by irreversibly binding to the 30S bacterial ribosome, thus inhibiting protein synthesis (Heijerman et al., 2009). The first formulation of tobramycin was approved in 1998 as tobramycin inhalation solution (TIS) (TOBI®, Novartis AG, Switzerland; Bramitob®, Chiesi Farmaceutici S.p.A, Italy) (Geller et al., 2011b). Since then, new dry powder formulations have been developed such as the tobramycin inhalation powder (TIP) (TOBI® Podhaler®, Novartis AG, Switzerland) that is the first marketed dry powder inhaled antibiotic. This dry powder has been manufactured using a technology, termed PulmoSphere® (Novartis AG, Switzerland) (VanDevanter and Geller, 2011), and has been shown to be even more effective as compared to TIS (Smyth et al., 2014; Hamed et al., 2017). Despite the fact that tobramycin is usually well-tolerated, some of the patients treated with TIP experienced cough, bronchospasm, moderate tinnitus and dysphonia (Heijerman et al., 2009; Shteinberg and Elborn, 2015). The approved dose for TIS is 300 mg of antibiotic nebulised by a jet nebulizer twice a day every other month (Cheer et al., 2003; Somayaji and Parkins, 2015), while for TIP the treatment consists in 112 mg (four 28 mg capsules) inhaled twice a day in alternating cycles of 28-day on/off treatment (Döring et al., 2012). These capsules are composed of low-density porous particles, whose absorption is improved using a portable DPI, named Podhaler® that improves flow and dispersion of formulation. The administration by inhalation of dry powder is preferred to aerosolized solution because it is simpler and shorter and significantly improves the quality of patient's daily life. Moreover, the recommended dose of TIP has showed a significant improvement in lung function compared to TIS (Gaspar et al., 2013).

##### Aztreonam Lysine

Aztreonam is an antibiotic belonging to the family of monocyclic β-lactams. It is active against Gram-negative bacteria by inhibiting the synthesis of their cell wall (Heijerman et al., 2009). The first formulation of intravenous aztreonam was developed in 1980s, and its excellent activity against PA and other pathogens made this antibiotic a promising drug for the treatment of chronic bacterial infections. However, several side-effects were registered in CF patients, such as rash, nausea, vomiting and diarrhea. Moreover, the intravenous formulation contained the arginine salt, which induced airway inflammation and caused bronchoconstriction. The substitution of arginine salt with lysine salt was subsequently made in order to develop a safer formulation of aztreonam for aerosolized use (Kirkby et al., 2011). Aztreonam lysine (AZLI) (Cayston®, Gilead Sciences Inc., USA) was approved in 2010 and the recommended dose is 75 mg inhaled thrice daily, in an alternate off/on cycle of 28 days. In several clinical studies it has been demonstrated that AZLI significantly improves clinical outcomes in CF, including lung function, quality of life, nutritional status and reduced bacterial density in sputum by efficaciously suppressing chronic PA infections (Döring et al., 2012; Heirali et al., 2019).

##### Colistin

Colistin, also known as polymyxin E, is a cyclic polypeptide antibiotic derived from *Bacillus polymyxa varietas colistinus*. Among the five different polymyxins discovered in 1947, only two of them have been used for clinical purposes, polymyxin B (known as colomycin) and polymyxin E (colistin) (Storm et al., 1977). The bactericidal action of colistin against gram-negative bacteria is given by its cationic nature (Heijerman et al., 2009). The cationic polypeptides of colistin interact with the anionic lypopolysaccharides in the outer membrane of the pathogen, causing an imbalance within the cell membrane and a subsequent change in permeability which ultimately causes cell death (Schwarz, 2015). Colistin was first marketed in the 1950s. Historically, it was administered intravenously, but reports of nephrotoxicity and neurotoxicity discouraged its use, especially after the appearance of other less toxic antimicrobials, such as tobramycin. However, the lack of treatment options for multi-drug resistant pathogens has led to the recovery of colistin as an antimicrobial agent, especially in its inhaled formulation (Lim et al., 2010). Polymyxin E is commercially available in two forms: colistin sulfate (for topic use) or as colistimethate sodium salt, an inactive prodrug given via intravenous or inhalation (Van Westreenen and Tiddens, 2010). While colistimethate sodium salt is currently used to treat chronic PA infection and is generally well-tolerated by inhalation, colistin sulfate is not suitable for treating CF patients due to several side effects, such as throat irritation, cough and severe bronchoconstriction (Gaspar et al., 2013). Colistin dry inhalation powder (CDPI) formulation is commercially available as Colobreathe® (Forest laboratories Inc., USA), which contains a dose of 125 mg colistimethate sodium salt to be administrated through a hand-held inhaler named Turbospin® (Forest laboratories Inc., USA). The recommended dose is a 125 mg capsule twice daily (Schuster et al., 2013). CDPI shows several advantages over the nebulized solution form, including shorter administration (1 min) and an enhanced portability (Conole and Keating, 2014). Colistin is often used in combination with other antimicrobials, such as ciprofloxacin and tobramycin, in order to improve the patient's conditions.

##### Fluoroquinolones

Fluoroquinolones belong to the family of quinolones, a group of molecules that is widely used as antibiotic because of their large spectrum of bactericidal activity, excellent bioavailability, rapid cellular uptake (Drusano, 1997; Wise and Honeybourne, 1999) and tolerance (Grillon et al., 2016). The bactericidal mechanism of action of fluoroquinolones consists in the inhibition of DNA replication and transcription by interacting with the DNA-gyrase complex (Young et al., 2013). This class of antibiotics are currently in a phase III of clinical trials (Maselli et al., 2017) as drugs to be administered during the “off” circle of TIS, in order to improve patient outcomes and to avoid drug resistance (Gaspar et al., 2013).

Ciprofloxacin and levofloxacin are the most investigated fluoroquinolones for the treatment of CF infections, due to their bactericidal activity against Gram-negative bacteria and their prolonged post-antibiotic effect (Andersson, 2003). Ciprofloxacin (first marketed as Cipro®, Bayer AG, Germany) is a second-generation fluoroquinolone. It is active against Gram-negative and Gram-positive bacteria, and it can be administered via oral or intravenous route, although inhalation formulations are under investigation and have reached the final phases of development. A phase II study on ciprofloxacin as dry powder for inhalation given at two doses levels (32.5 and 48.75 mg) twice daily for 28 days, has showed a significant decrease in PA density compared to placebo (Döring et al., 2012). The main side effects of ciprofloxacin are cartilage toxicity, sunlight sensitivity rash and emergence of resistance.

Levofloxacin is a third-generation fluoroquinolone with higher activity against Gram-positive pathogens compared to ciprofloxacin (Young et al., 2013). It can be administered either orally and intravenously, and recently also via inhalation with a novel liquid formulation (MP-376 or Aeroquin™, Mpex Pharmaceuticals, Inc., USA), that has reached the phase II study (Geller et al., 2011a). Aeroquin™ showed a reduced need for other antibiotics against PA for different doses given, which are 120 or 240 mg every day, and 240 mg twice a day for 28 days, respectively. Aeroquin™ exhibit a good degree of tolerance in patients treated with the antibiotic with respect to placebo, and is currently being studied in phase III of clinical trials (Ballmann et al., 2011; Döring et al., 2012).

##### Fosfomycin

Fosfomycin (first marketed as Monuril™, Zambon S.p.A, Italy) is a broad-spectrum antimicrobial active agent against Gram-negative and Gram-positive bacteria, and it is particularly effective against multidrug-resistant PA (Mirakhur et al., 2003; Spoletini et al., 2018). This drug exhibits bactericidal activity by inhibiting the initial step in cell wall synthesis, and it is commercially available in intravenous formulation as fosfomycin disodium, or in oral formulation as fosfomycin disodium and fosfomycin trometamol (Van Westreenen and Tiddens, 2010; Gaspar et al., 2013). Due to the easy development of drug resistance when the same antibiotic is administered repeatedly, fosfomycin is often used in combination with other antibiotics in order to prevent cross-resistance (Trapnell et al., 2012). Studies have shown that the intravenous administration of fosfomycin combined with other antimicrobials to patients colonized by multi-resistant PA resulted in a general improvement of the patients status with low side effects (Mirakhur et al., 2003). A combination of fosfomycin and tobramycin in a 4:1 ratio (FTI, Gilead Sciences Inc., USA) has reached phase III study, in which the safety and efficacy of FTI treatment followed by a 28 days course of AZLI has been evaluated. These studies demonstrated an improvement of clinical conditions in CF patients infected by PA. Moreover, the preliminary results obtained from this research suggest that fosfomycin could be used not only for the treatment of PA, but also for the treatment of other pathogens detected in the CF population (Ballmann et al., 2011; Döring et al., 2012).

#### Anti-inflammatory Drugs

CF patients often suffer from chronic airway inflammation, which ultimately leads to several physiological and metabolic changes, including weight loss, anorexia and metabolic breakdown. Hence, anti-inflammatory therapy represents an additional option to antibiotic treatments to avoid a decline in lung function (Heijerman et al., 2009). Several clinical trials have proven that anti-inflammatory therapies have positive outcomes; however, the related side effects have limited the use of these drugs for CF treatments (Konstan and Davis, 2002). The first anti-inflammatory drugs studied in CF were systemic corticosteroids and high-dose ibuprofen (Ibrahim et al., 2011b). Corticosteroids are used in CF to reduce endobronchial inflammation, but they also produce increases in the incidence of diabetes, cataracts, and growth failure. New inhalation formulations of these drugs have been developed in order to minimize systemic adverse side effects, although one potential disadvantage of inhaled corticosteroids is their difficulty in penetrating the sticky mucus within the CF patient's airways (Dinwiddie, 2005).

Compared to corticosteroids, the non-steroidal ibuprofen offered promising results in CF patients. Ibuprofen has been studied as a treatment for CF due to its ability to inhibit the migration and activation of neutrophils (Ramsey, 1996). These studies also demonstrated the effective benefit of ibuprofen therapy on CF patients, especially when the treatment is started before the development of severe inflammation and pathological changes in the lung (O'Sullivan and Freedman, 2009) and in children 5–13 years old with mild lung disease. The main disadvantage of the ibuprofen therapy is related to its narrow therapeutic window, which results in a particular carefulness in drug dosage; in fact, while low concentrations might not be sufficient to inhibit neutrophils migration in the lung, high dosage leads to an increased risk of gastrointestinal and renal toxicity (Turcios, 2005).

Other molecules primarily employed as antibiotics or mucolytics show ancillary anti-inflammatory activities with positive effects on CF treatment. As an example, studies carried out on oral administration of azithromycin, an antibiotic belonging to macrolides also employed as anti-inflammatory drug, have demonstrated an improvement of lung function (Friedlander and Albert, 2010). Leukotriene modifiers, oral N-Acetylcysteine, and inhaled glutathione are other kinds of anti-inflammatory drugs that were tested for CF. However, the CF Foundation has determined that there is not enough evidence to recommend for or against these drugs in patients with CF (Dasenbrook and Chmiel, 2017). Immunoglobulins and dornase alpha (DNase) (Sepe et al., 2019) are also used as anti-inflammatory agents in addition to their mucolytic activity.

#### Mucolytics and Bronchodilators

As reported above, CF airways are characterized by the presence of a thick, viscous mucus. Hence, mucolytics and bronchodilators are often administered to improve airway clearance (Ong et al., 2019). Two main muco-active agents are administered via aerosol: N-acetylcysteine (NAC), which disrupts disulphide bonds in mucus, and DNase (Pulmozyme®, Genentech), which acts on enzymatically break down DNA in airway secretions. NAC lowers mucus viscosity and elasticity by substituting sulfhydryl groups for the disulphide bonds connecting mucin proteins. Differently, DNase mucolytic activity consists in its ability to reduce the size of DNA released in the sputum. In infected airways, in fact, the degeneration of neutrophils causes the release of DNA, which further increases the viscosity of secretions (Henke and Ratjen, 2007). In 2014 Charrier et al. developed a new cysteamine-based mucoactive agent with antimicrobial and antibiofilm features, marketed as Lynovex® (Novabiotics ltd., UK). This drug, which is already used for the treatment of cystinosis, has successfully completed a phase IIb clinical trial for oral formulation in acute infectious CF exacerbations, with positive top line data reported. Clinical studies are expected in 2019 (Charrier et al., 2014).

β-adrenergic agents, such as salmeterol, are the most commonly prescribed bronchodilators (Halfhide et al., 2016). They act by broadening the airways and relaxing airway muscles, then making easier to breathe. Other bronchodilators currently in use are anticholinergics.

### CFTR Modulators

Since the identification of the mutations in the gene coding the CFTR protein, several new drugs were developed to target the disease at the CFTR level by improving the defective or deficient activity of the mutated protein. Recently, a new and promising class of drugs called CFTR modulators has entered the CF therapeutic landscape. These drugs differ from prior therapies in that they aim to improve the production, intracellular processing, and/or function of the defective CFTR protein. Currently, five types of CFTR modulators have been developed: (i) read-through agents, which rescue protein synthesis; (ii) correctors, which enhance conformational stability of CFTR, thus rescuing the trafficking of the mature protein to the plasma membrane (PM); (iii) potentiators, which improve CFTR channel activity; (iv) stabilizers, which anchor CFTR channel at the PM and decrease protein degradation rate, thereby correcting the instability; (v) amplifiers, which work by increasing the amount of CFTR protein on the cell surface providing additional substrate for correctors and potentiators to act upon (Lopes-Pacheco, 2016). Several clinical trials are examining all these different classes to increase or restore the activity of the mutated CFTR protein in patients (Clancy et al., 2019).

Besides the general overlook of the main CFTR modulators reported in this section, many other therapeutic agents based on correctors and potentiators are undergoing various pre-clinical and clinical studies, and are extensively described by Rafeeq and Murad (2017).

#### CFTR Read-Through Agents and Amplifiers

CFTR amplifiers are novel mutation-independent compounds that work by increasing immature CFTR protein expression by stabilizing CFTR mRNA. These amplifiers, that act synergistically with correctors and potentiators to augment therapeutic benefits, can effectively overcome any “substrate” limitation. There are two types of amplifiers which have showed promising results in pre-clinical and clinical trials, PTI-428 and PTI-CH (Proteostasis Therapeutics Inc., USA), even though they are not yet available on the market (Kym et al., 2018; Haq et al., 2019). Read-through agents could benefit CF patients affected by class I mutations, since this mutation causes the presence of a premature stop codon which precludes the synthesis of full-length CFTR protein. Among read-through agents, Ataluren (also known as PTC124, PTC therapeutics Inc., USA) emerged as a mutation-based treatment for approximately 10% of patients bearing non-sense premature stop mutations that disrupt the production of full-length functional CFTR. Ataluren act by inducing premature termination codon (PTC) read-through and hence restores CFTR expression at the PM. In a phase II clinical trial Ataluren showed an improvement in the forced expiratory volume in 1 s (FEV_1_), although subsequent trials confirmed a slight improvement in FEV_1_ only in a subgroup of patients (Tosco et al., 2018). Other read-through agents have shown promising results in preclinical trials when combined to ivacaftor, including Ataluren derivatives and the FDA-approved natural herbal agent, escin (Mutyam et al., 2016).

#### Ivacaftor

Ivacaftor (Kalydeco™, Vertex Pharmaceuticals Ltd, UK), also known as VX-770, is a CFTR potentiator that increases CFTR channel opening on apical cell membrane (Thompson, 2016), allowing chloride ion to flow through the CFTR protein on the surface of epithelial cells. It has been used initially to restore CFTR function for G551D, the most common class III mutation, although its efficacy has been demonstrated for other less common mutations (such as G1244E, G1349D, G178R, G551S, S1251N, S1255P, S549N, and S549R) (Ponzano et al., 2018; Tosco et al., 2018). Being approved by FDA in 2012, Ivacaftor is the first commercially available treatment to target the basic defects in CF (Pettit, 2012). The efficacy and safety of Ivacaftor has been proved by several studies (Ramsey et al., 2011; De Boeck et al., 2014), where it was demonstrated a meaningful increase in FEV_1_ and a reduction in sweat chloride to a value below the usual diagnostic threshold for CF. Ivacaftor is generally well-tolerated by patients. The most occurring complication is represented by an elevation of liver enzymes, whose levels, however, return to baseline after drug discontinuation (Haq et al., 2019).

#### Lumacaftor

Lumacaftor (Vertex Pharmaceuticals Ltd, UK), also known as VX-809, has been approved by FDA in 2015 as a CFTR corrector for class II mutations, especially the F508del. This molecule improves CFTR maturation, thus allowing the protein to reach the cell membrane and transport chloride ions. In a large number of *in vitro* studies, Lumacaftor showed good efficacy and tolerability, although the treatment by itself showed a significant decrease only in sweat chloride levels and no improvements in FEV_1_ in a phase II trial with F508del-homozygous patients. In order to reach greater effects, accurate studies were carried out in combinations with correctors; compared to monotherapies, the combined therapy offers an enhancement of the CFTR rescuing in patients bearing F508del, as well as other II class mutations.

#### Orkambi™ and Symdeko™

Due to the multi-domain structure and folding of CFTR, a single modulator is usually insufficient to act positively on different protein misfoldings. Despite an increased CFR modulators availability, ~40% of patients with CF are without effective therapy. To overcome this limitation, newer modulator-based therapies are made of a combination of drugs (Rafeeq and Murad, 2017). The first double formulation, consisting in a combination of Ivacaftor and Lumacaftor (Orkambi™, Vertex Pharmaceuticals Ltd, UK), have been approved in 2015 by FDA and EMA for patients who are homozygous for the F508del CFTR mutation. It has been found to be safe and effective, with a significant improvement in FEV_1_ and a reduction in exacerbations (Wainwright et al., 2015). The combination of Tezacaftor and Ivacaftor (Symdeko™ Vertex Pharmaceuticals Ltd, UK) is currently in phase III of clinical trials, and although it shows weak effectiveness in patients with F508del paired with another minimal function CFTR mutation, it has demonstrated to be more effective in patients homozygous for F508del or other heterozygous F508del individuals (Paranjape and Mogayzel, 2018).

#### Triple Therapy

Different combinations of CFTR modulators, along with the simultaneous administration of different drugs, are the key points of the precision medicine. This emerging paradigm, which combines preventive and therapeutic strategies, takes into account differences among individuals, and focuses on a patient-centered rather than mutation-centered approach (Maiuri et al., 2017). The newer and most promising research involves the use of first-generation potentiators and correctors in combination with a next-generation drugs, such as VX-152, VX-440, VX-445, and VX-659 (Vertex Pharmaceuticals Ltd, UK). This therapeutic approach, called triple therapy, has shown promising results for the restoration of the most common CFTR mutations. A phase II study of VX-440 in combination with Tezacaftor and Ivacaftor in CF patients having one F508del mutation and one minimal function mutation, as well as patients with two copies of the F508del mutation, reported an efficacy in the range of 9.6–12% improvement in FEV_1_ at the highest dosage in trials ranging from 2 to 4 weeks for both F508del homozygous and heterozygous patient populations (Chaudary, 2018; Haq et al., 2019). Following these positive responses, new formulations are currently under development. Recently, a new combination therapy has been approved by FDA as Trikafta™ (Vertex Pharmaceuticals Ltd, UK). It combines the action of two correctors (Elexacaftor and Tezacaftor) and a potentiator (Ivacaftor). This triple therapy showed increased therapeutic effects with respect to Orkambi™ or Symdeko™. In a randomized phase 3 study, Taylor-Cousar et al. developed a triple combination of next-generation correctors (VX-659 and VX-445) with Tezacaftor and Ivacaftor for patients with one or two F508del CFTR alleles. The results obtained showed an enhanced therapeutic activity that triple combinations provide in patients with F508del/F508del genotype (Taylor-Cousar et al., 2019).

#### CFTR Modulators That Act by Regulating Proteostasis and Autophagy

Recent evidences indicate that CFTR does not act only as ion chloride channel, but is involved in the epithelial stress response, influences the proteostatic network (the cellular processes which regulate the fate of a protein inside the cell) and modulates autophagy. Autophagy is the most important mechanism for protein turnover and is essential in promoting cellular clearance from protein aggregates and ROS sources. Defective autophagy and impaired cellular proteostasis leads to a worsening of chronic CF lung disease. Thus, novel intervention strategies aim to correct the underlying proteostasis and autophagy impairments by rescuing F508del-CFTR to the PM. Lately, studies on proteostasis modulators and autophagy inducers reported encouraging results in preclinical phases (Bodas and Vij, 2019). Cysteamine, a proteostasis regulator FDA-approved 30 years ago for nephropathic cystinosis, has shown promising results in CF treatment (Charrier et al., 2014). Marketed in oral formulation as Cystagon™ (Recordati S.p.A, Italy) and Procysbi™ (Horizon Therapeutics plc, Ireland), it acts by targeting transglutaminase 2 (TG2, a multifunctional intermediary in cell autophagy). The TG2 inhibition increases the expression of CFTR and restores its function. A phase II trial of cystamine in combination with epigallocatechin gallate has been completed in homo and heterozygous CF patients with F508del (De Stefano et al., 2014; Tosco et al., 2016). The results showed decreased chloride concentrations, increased CFTR function, autophagy restoration and improvement in FEV_1_.

### Gene Therapy

For many years, the main CF treatment strategies were focused on the downstream effects of CFTR dysfunction, such as airway obstruction, infection and inflammation. However, after the identification and cloning of the gene encoding the CFTR in 1989, along with an increased knowledge on how CFTR dysfunction causes lung disease, new targets and approaches for the CF treatment have been investigated (Ratjen, 2009). Hence, the novel strategies aim to restore or replace the CFTR gene rather than treating the pathologies associated with its defect (d'Angelo et al., 2014; Davies et al., 2019).

**Figure 3:**
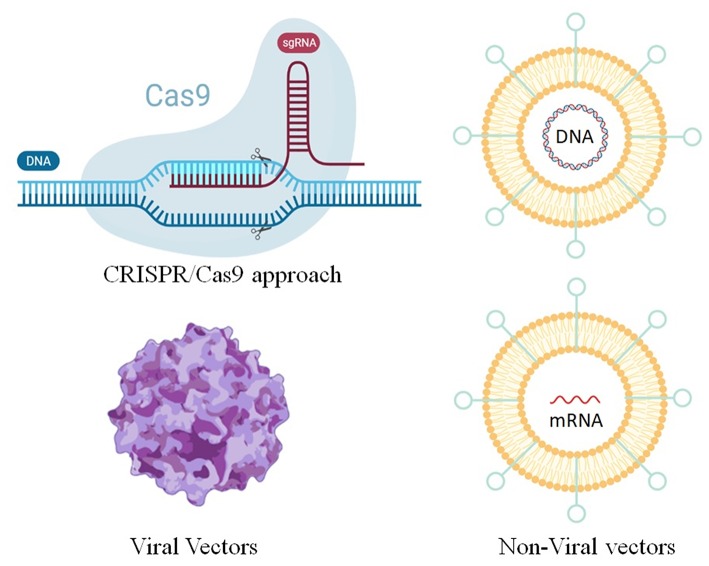
Methods for gene editing and therapy for the treatment of CF.

In general, gene therapy consists in delivering nucleic acids into a cell to replace or repress genes or biological functions. Due to its monogenic and autosomal nature, CF was one of the first diseases considered for this approach (Ibrahim et al., 2011b; Savla and Minko, 2013). In fact, in this pathology, the CFTR gene is the only gene affected, and the correction of only one of the two alleles is sufficient to revert the disease (Villate-Beitia et al., 2017). Furthermore, a small increase in CFTR expression may be beneficial to CF patients, since studies has shown that even in normal patients the protein is not expressed in high amounts (20–100 CFTR protein/cell). Several vectors have been tested and among these, viral systems are the most widely used, although non-viral vectors recently emerged as safer, easier and cheaper options with respect to viral vectors (Smith, 1997; Ratjen, 2009) (Figure 3). Finally, the CRISPR/Cas9 approach which is emerging as a powerful tool for engineering the genome in diverse organisms, is another interesting method for the gene theory of CF (Figure 3).

#### Viral Vectors

Adenoviral vectors (AV) were the first systems used for gene therapy clinical trials in 1993 by Zabner et al. (1993). The aim of this study was to evaluate the correction of the chloride transport defect, characteristic of CF-affected epithelia, by using an E1-deficient adenovirus, which expresses CFTR protein in airway epithelial cells. The target organ was the nasal epithelium, since the composition of this latter is similar to the lower airways but of much easier access. The results obtained by testing the therapy in three patients showed an effective correction of the chloride transport defects, without evidence of severe side effects. Even though the low number of treated subjects made difficult to establish the real efficacy of this approach, this study paved the way for subsequent investigations on AV for the treatment of CF. In fact, in the following years, more AV trials were carried out showing a partial efficacy of the therapy which was, however, lower than originally predicted by the pre-clinical trials (Griesenbach and Alton, 2009, 2013). Moreover, this transduction efficiency in most tissues was counterbalanced by adverse effects, such as inflammatory responses in the host organism. Hence, other gene delivery systems have been developed, such as adeno-associated viral vectors (AAVs).

AAVs exhibit several features suitable for gene delivery, such as high transduction efficiency, and broad serotype-dependent tissue tropism. Although a large number of serotypes have been identified, the most extensively investigated is the serotype AAV2. Several studies were carried out in which the vector was administered to the nose, sinuses and lungs of CF patients. Moss et al. (2004) confirmed the safety and tolerability of repeated doses of aerosolized AAV2 containing CFTR complementary DNA (cDNA). However, further studies on a larger number of patients showed a lack of improvement in lung function for repetitive administration (Moss et al., 2007). One of the possible reasons of these discouraging results may be attributed to the limited packaging of AAV, resulting in a low expression of the CFTR cDNA. Although AAVs present some disadvantages, arising mainly from the use of viruses as carriers, the ongoing research is trying to address and improve these limitations and develop novel and safer formulations (Griesenbach and Alton, 2012; Guggino and Cebotaru, 2017).

#### Non-viral Vectors

Non-viral vectors have the potential to overcome some limitations of viral vectors, due to their easy scale-up processing, safety and stability. They are constituted of two components, the nucleic acid (which, for CF, is a plasmid carrying the CFTR cDNA) and a carrier molecule which binds to the DNA. The recent advances in the field of material science and nanotechnology have prompted the development of nanomaterials which can be used as carriers for non-viral gene transfer formulations (Villate-Beitia et al., 2015, 2017). The two main classes of nanocarriers currently employed in non-viral vectors, cationic lipids and cationic polymers, act similarly by binding to negatively charged DNA. These classes of nanocarriers will be discussed in detail within section Nanotechnology for CF Treatment.

#### CRISPR/Cas9 Approach

The CRISPR/Cas9 approach (Clustered Regularly Interspaced Short Palindromic Repeats) is a gene-editing strategy in which the specific mutated sequence of the defective CFTR gene is corrected by introducing changes in the DNA, allowing a permanent correction of genetic defects underlying disease (Pranke et al., 2019). This system is based on the bacterial CRISPR immune system, which confers resistance and adaptation. CRISPR are DNA sequences composing bacteria's genomes derived from DNA fragments of viruses that have infected the prokaryotic cell, which allow to prevent subsequent infections of the host organism (Doudna and Charpentier, 2014; Barrangou, 2015). Cas9 (CRISPR-associated protein 9) is an enzyme that uses CRISPR sequences as a guide to recognize and cleave specific strands of DNA complementary to the CRISPR sequence (Brouns et al., 1993; Ran et al., 2013). Engineered Cas9 cleaves the DNA in a sequence-specific mode and creates a specific double-stranded break, so the cell can fill the excised portion with the correct gene sequence. One of the most promising applications of CRISPR/Cas9 is given by its possible use as a technology for treating genetic disorder, including CF. This approach has been already tested in canine models for the treatment of Duchenne Muscular Dystrophy (Amoasii et al., 2018; Lim et al., 2018), and despite it has not been tested in animal models for the treatment of CF, it has been found effective in correcting CFTR defects (Hsu et al., 2014). The first studies on CRISPR/Cas9 on CF were carried out in 2013 by Schwank et al. (2013), in which they tested the recovery of functional CFTR in intestinal organoids obtained from CF patients, and proved the repairing of the mutation at the CFTR locus with CRISPR/Cas9 gene editing. More studies were carried out to investigate the potential of this gene editing approach, even though there are concerns about its applicability *in vivo* directly to the lungs: in fact, since Cas9 has bacterial origins (mainly *Staphylococcus aureus*) it is potentially immunogenic especially in CF patients whose lungs are colonized by this pathogen. Moreover, CRISPR/Cas9 can be destroyed by the cellular immune response, thus reducing its therapeutic effect (Miah et al., 2019).

## Nanotechnology for CF Treatment

The rapid progress of nanomedicine creates new perspectives to enhance the efficacy of inhalation treatment for lung diseases. In general, the application of nanotechnology to the design of drug delivery systems mainly allows a more effective delivery of therapeutics within the target site, thus preventing several adverse side effects of tissues and cells. Other important advantages include the achievement of enhanced therapeutic effects with lower drug doses, and the possibility to deliver hydrophobic molecules while ensuring the protection of the compounds from degradation (da Silva et al., 2017). Nano-sized structures, such as nanoparticles (NPs) and nanodevices, exhibit peculiar physical, chemical and biological properties (Zhang et al., 2010). In addition, nano-sized structures have dimensions comparable to those of biological molecules such as proteins and carbohydrates, hence they can easily interact with them inside and outside cells (Bahadori and Mohammadi, 2012). Drugs can be delivered to target sites either as entrapped or encapsulated within the NPs structure, or as adsorbed/attached on the NPs surface (Sung et al., 2007).

In the case of CF, the use of NPs could be a valid approach to overcome the thick mucus layer that is formed within the alveolar region as well as the bacterial biofilm (Ong et al., 2019). The CF mucus contains less water than normal mucus and presents mucin fibers (70–80%), DNA, actin and other macromolecules, which form a cross-linked network where the mesh size is reduced from above 500 nm (in healthy patients) to 300–100 nm in CF patients, depending on their conditions and on the disease stage (Ensign et al., 2012; Liu et al., 2014; Leal et al., 2018). This significant change in mucus structure and composition leads to an increased viscoelasticity (Ibrahim et al., 2011b; Ong et al., 2019), along with the formation of hydrophobic and electrostatic barriers that strongly inhibit NPs penetration. Small NPs are able to diffuse through the network of the mucus while larger ones are trapped in the meshes by physical size exclusion effect. However, penetration strongly depends also on NPs surface properties that influence the interaction with mucus such as superficial charge and hydrophobicity. It was demonstrated that the interaction between NPs and the mucus can be also decreased by modulating the particle surface charge, which needs to be as neutral as possible (with ζ-potential values in the ±10 mV range). Positively charged NPs tend to interact electrostatically with the mucins and the abundant amounts of free DNA and filamentous actin that are all negatively charged, while negatively charged NPs are repelled from the mucus by the hydrophobic domains on the mucins. Thus, neutral particles show good permeation properties due to lacking electrostatic interactions. Two common strategies to overcome the CF mucus barrier are the coating of NPs surface with a dense layer of the so-called “muco-inert” polymers [i.e., low-molecular-weight polyethylene glycol (PEG)] to eliminate both electrostatic and hydrophobic interactions or the use of mucolytic agents [i.e., N-acetylcysteine (NAC)] to reduce mucosal viscoelasticity (Savla and Minko, 2013; Ong et al., 2019).

Despite all the advantages of a NPs mediated therapy for the treatment of pulmonary disease, it is worth to highlight that the development of NPs formulations suitable for administration to the lungs by inhalation is not straightforward due to the complexity of lung anatomy and physiology and to NPs deposition mechanisms.

In order to be effective, when inhaled NPs must reach the deep airways and penetrate the mucus covering the alveoli. Hence, their dimension is a fundamental parameter to be considered in order to predict their deposition. The size of NPs must be small enough to penetrate the mucus and meanwhile big enough to avoid rapid exhalation. It has been proved that the optimal range that allows a good deposition in the alveolar region of the lungs is between 1 and 3 μm (Sung et al., 2007) (in which NPs deposit with gravitational sedimentation) and below 500 nm (in which NPs deposit in the deeper lung by a Brownian diffusion). Besides NPs size, however, there are other parameters that affect the deposition efficacy, such as particle morphology and the surface properties (Paranjpe and Müller-Goymann, 2014).

Nebulization can be used to overcome some NPs-to-lung delivery issues. Nebulizers generate aerosol from solutions or suspensions and create droplets with appropriate size for pulmonary delivery. However, the biggest limitation of this approach arises from the difficulty of developing long term stable nano-suspensions in order to avoid NPs aggregation and degradation. Moreover, most nebulizers show practical disadvantages from patient's point of view, such as limited portability, time-consuming application, low efficiency and high bacterial contamination. Hence, dry powder formulations are usually preferred (Newhouse et al., 2003). A well-known approach for the administration of NPs with DPI consists in the formation of nano-embedded microparticles (NEMs). These nano-in-micro systems [also called Trojan particles (Tsapis et al., 2002)] are able to transport and efficaciously release NPs within the lungs after the dissolution of the microparticle matrix, allowing NPs penetration within the mucus barrier. The most common technique used to convert NPs suspensions into stable, inhalable microparticles is the spray drying (Ruge et al., 2016). Recent studies have highlighted how the tuning of process parameters and the use of excipients can ensure a good re-dispersibility of NEM powders (Tomoda et al., 2008; Kho and Hadinoto, 2010). Several researches have focused on NEMs approach; the most widely used materials are biocompatible and biodegradable polymers as PLGA (Lebhardt et al., 2011; Ungaro et al., 2012b), chitosan (Grenha et al., 2005), gelatin (Sham et al., 2004), PCL (Kho et al., 2010), and polyacrylate (Hadinoto et al., 2007), usually coupled to mannitol, lactose or cyclodextrins as excipients.

The template-assisted technique is another method to prepare NEMs. In this approach, the cylindrical pores of a membrane serve as templates for the formation of elongated NPs, which can be released upon membrane's dissolution. Despite the template-assisted deposition is specific for conducting materials, which are usually insoluble in water and thus are difficult to be employed for drug delivery applications, it represents an innovative and promising approach to prepare NEMs with elongated shape, which are expected to better deposit in deeper lung regions than spherical NPs (Kohler et al., 2011; Tscheka et al., 2015).

**Figure 4:**
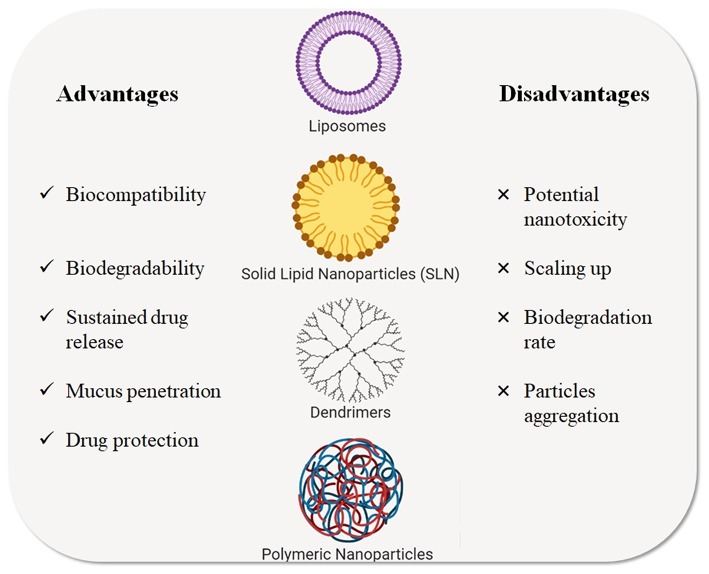
Types of nanotechnological platforms currently used for CF treatments. Advantages and disadvantages are highlighted.

Over the last decade, different types of NPs have been studied as nanocarriers for gene and drug delivery in CF treatment. Among these, liposomes, polymeric NPs, solid lipid NPs, and dendrimers (Zhang et al., 2010) represent the major classes of NPs for inhalation therapy that have been tested for CF treatment and that are described in the next paragraphs (Figure 4) and the listed in Table 2.

**Table 2:** Examples of nanotechnological formulations for the CF treatment.

**Nanocarrier**	**Composition/material**	**Drug**	**Key finding**	**References**
Liposomes and Lipoplexes	DPPC/Chol	Polymyxin B	Increased bioavailability and bactericidal activity against PA	Omri et al., 2002
	DSPC/DMPG	Tobramycin	Increase of drug persistence *in situ* and higher local concentration	Omri et al., 1994
	DSPC/DMPG	Tobramycin	Increased drug activity *in vivo* when encapsulated in fluid liposomal formulation compared with free drug and drug-loaded rigid liposomes	Beaulac et al., 1996, 1997
	DSPC/DPPC			Sachetelli et al., 1999, 2000
	DSPC/DPPC			
	DPPC/DMPG			
	DPPC/Chol	Amikacin	Improved penetration within PA biofilm	Meers et al., 2008; Okusanya et al., 2009
	PC/DOPE/SA	Meropenem	Higher drug efficacy when encapsulated within cationic liposomes	Drulis-Kawa et al., 2006
	PC/DOTAP/Chol			
	DPPC/Chol	Amikacin Gentamicin Tobramycin	Improved drug efficacy when encapsulated within cationic liposomes	Mugabe et al., 2006
	DSPC/Chol	Amikacin Tobramycin	Enhanced antibiotic penetration into the bacteria cell membranes	Halwani et al., 2007
	PC/Chol/DOTAP PC/DOPE/DOTAP	Gentamicin Ciprofloxacin Meropenem	Increased drug activity for lower concentration administered and better drug penetration within bacterial strains	Gubernator et al., 2008
	DMPC/Chol	Gentamicin	Better pseudomonal activity compared to free drug	Rukholm et al., 2006
	DPPC/Chol POPC/Chol	Polymyxin B	Better drug penetration and efficacy within bacterial cells	Alipour et al., 2008
	EPC/Chol	Budesonide	Increased drug persistence in the lungs	Joshi and Misra, 2001
	PC/Chol/DSPG	Amikacin	Prolonged drug persistence and activity	Fielding et al., 1998
	PC/Chol/DSPE	Gentamicin	Increased therapeutic efficacy, increased survival rate of rates	Schiffelers et al., 2001
	DPPC, DOPC, DPPG	Tobramycin	Better drug penetration when encapsulated in cationic liposomes, increased drug efficacy	Messiaen et al., 2013
	DOTMA/DOPE	siRNA	Efficient restoring of mucus hydration and airway clearance	Manunta et al., 2017
	DOTMA/DOPE	siRNA	Effective correction of mucociliary defects	Tagalakis et al., 2018
	GL76A	pGM169	Increase in FEV_1_ and lung function stabilization	Alton et al., 2015
	DC-Chol/DOPE	CFTR cDNA	Partial restoration of Chloride secretion	Alton et al., 1993; Middleton et al., 1994
	DOTAP	pCMV-CFTR	Effective gene transfection with no side effects	Porteous et al., 1997
	DOPC/Chol	Colistin	Good stability of liposome/drug complex	Wallace et al., 2012
Solid Lipid Nanoparticles	SA/PC	Myriocin	Significant reduction of lung inflammation	Caretti et al., 2014
	Chol/lecithin	Tobramycin	Better drug deposition within the lungs	Pilcer et al., 2006, 2008
	DSPC	Ciprofloxacin	Reduced adverse effects compared to oral and intravenous ciprofloxacin	Stass et al., 2013a,b
	DMA/DSPC/Chol/DMG	cmCFTR	Positive CFTR restoration	Robinson et al., 2018
	GMS	Budenoside	Good drug dispersion within the Lipid matrix	Zhang et al., 2011
	Albumin Gelatin Chitosan	Ciprofloxacin	Sustained and controlled drug release	Jain and Banerjee, 2008
		Amikacin	Higher drug concentration in the lungs with lower side effects	Varshosaz et al., 2010, 2013; Ghaffari et al., 2011
Dendrimers	PAMAM G4	siRNA	Excellent cellular uptake and gene silencing	Conti et al., 2014
				Bielski et al., 2017
				Agnoletti et al., 2017
	PAMAM-DEN^CYS^	Cysteamine	PA infection and growth reduced and rescue of CFTR protein	Brockman et al., 2017
Polymeric nanoparticles and microparticles	PLGA	Curcumin	Improved drug bioavailability and efficacy compared to free drug	Cartiera et al., 2010
	PLGA/PEG	PS-341	Sustained and more effective drug release and penetration	Vij et al., 2010
	PLGA	Ciprofloxacin	Drug antimicrobial activity and improved mucus penetration	Günday Türeli et al., 2017
	PEG/PLGA	Tobramycin	Improved mucus penetration, enhanced antimicrobial activity	Ernst et al., 2018
	PLGA/chitosan	cmRNA	Reduced chloride secretion and restoration of lung functions	Haque et al., 2018
	PVA-Alg/PLGA Chitosan-Alg/PLGA	Tobramycin	Deposition of NPs in the lungs depends on NPs size and composition	Ungaro et al., 2012a
	PGA/PLGA Dextran/PLGA	DNase	Enhanced mucolytic activity on CF sputum	Osman et al., 2013
	PLGA	Plasmid DNA	PLGA-mediated gene transfer can produce prolonged gene expression, despite gene transfer efficiency must be improved	Stern et al., 2003
	PLGA	dec-ODN	Inhibition of NF-κB transcriptional activity and reduction of chronic lung inflammation	De Stefano et al., 2011, 2013
	PEI Chitosan	miRNA	Better efficiency of PEI in facilitating miRNa uptake compared to chitosan	McKiernan et al., 2013
	PEI	Plasmid DNA	Improved penetration and transport in CF mucus, reduced activity due to aggregation	Ibrahim et al., 2011a
	PEG/PEI	Plasmid DNA	Efficient mucus penetration, improved distribution and retention of NPs, enhanced gene transfer and delivery without significant toxicity	Suk et al., 2014
	Polixamines	mRNA plasmid DNA	Enhanced mRNA and pDNA expression without exhibiting toxicity	Guan et al., 2019

*DPPC, dipalmitoyl-phosphatidylcholine; Chol, Cholesterol; DSPC, distearoylphosphatidylcholine; DMPG, dimyristoyl-phosphatidylglycerol; PC, phosphatidylcholine; DOPE, dioleoyl-phosphatidylethanolamine; SA, stearylamine; DOTAP, dioleoyltrimethylammonium propane; DMPC, dimyristoylphosphatidylcholine; EPC, egg phosphatidylcholine; POPC, palmitoyloleoylphosphatidylcholine; DOPC, dioleoylphosphatidylcholine; DPPG, dipalmitoylphosphatidylglycerol; DSPE, istearoyl-sn-glycero-phosphoethanolamine; DSPG, distearoyl-sn-glycerophosphoglycerol; DOTMA, dioleyloxypropyl-trimethylammonium chloride; GL67, Genzyme lipid; PEG, poly(ethlenglycol); PEI, poly(ethylenimine); PLGA, poly(lactic-co-glycolic acid*.

### Liposomes

Liposomes, whose structure was first described in 1965, are spherical lipid vesicles with size ranging from 20 nm to 20 μm, formed by a bi-layered membrane consisting of amphiphilic lipid molecules. Depending on their structure, liposomes can be classified in unilamellar vesicles (formed by a single bilayer membrane, size range from 25 nm to 1 μm) or multilamellar vesicles (constituted of several bilayer membranes, size range from 0.1 to 20 μm) (Rudokas et al., 2016). Due to their amphiphilic nature, lipid vesicles can entrap both lipophilic (within the bilayer) and hydrophilic drugs (within the aqueous core) (Moreno-Sastre et al., 2015). Liposomes have been extensively tested as inhalation delivery systems for many drugs, vitamins and nucleic acids for several diseases and pathological conditions (Kuzmov and Minko, 2015). One of the greatest advantages of these carriers is given by the design versatility that allows to obtain structures with the desired size and properties.

Antibiotic-loaded liposomes have been extensively studied to improve their pharmacokinetics and bioavailability, decrease their toxicity and achieve target selectivity (Drulis-Kawa and Dorotkiewicz-Jach, 2010). Liposomal antibiotics have the important advantage to overcome bacterial drug resistance of extracellular pathogens, along with their ability to protect the antibiotic molecules from enzymatic inactivation and degradation (Alipour et al., 2009). Lipid composition, size, charge, and drug/lipid ratio all influence the drug carrying capacity, the release rate and the lung deposition of liposomes (Cryan, 2005). Some phospholipids currently used as drug delivery systems are phosphatidylcholines (PC), phosphatidylglycerols (PG), phosphatidylethanolamines (PE), phosphatidylinositols (PI), dipalmitoyl-phosphatidylcholine (DPPC), distearoylphosphatidylcholine (DSPC), dimyristoyl-phosphatidylglycerol (DMPG), dimyristoyl-phosphatidylcholine (DMPC), and dioleoyl-phosphatidylethanolamine (DOPE) (Cryan, 2005; d'Angelo et al., 2014).

Liposomes can be prepared for inhalation both in liquid and in dry formulations, and several researches have proved their efficacy and safety when administered by inhalation. Some of the first studies on liposomal formulations were carried out in the 90s by Omri et al., who investigated the redemption (Omri et al., 1994), efficacy and toxicity (Beaulac et al., 1996, 1997) of liposome-encapsulated tobramycin in a comparative study on healthy and PA infected rats. The results showed a significant increase in drug residence time for both healthy and infected rats as compared to free tobramycin administration. The effects of liposomal composition on the drug release kinetics and efficacy were evaluated by the development of the so-called fluidosomes® [liposomes composed of DPPC: DMPG (10:1 to 15:1 ratio)] loaded with tobramycin. The administration of tobramycin-loaded fluidosomes in infected rats led to a complete eradication of the pathogen as compared to solid liposomes and free antibiotic. Furthermore, the detection of low levels of drug in the kidneys after fluidosomal administrations compared to free tobramicyn suggested a potential reduced nephrotoxicity. Drulis-Kawa et al. (2006) tested several liposomes loaded with meropenem (a β-lactame antibiotic) against PA strains, and demonstrated an improved efficacy of the drug loaded in two different cationic liposomes (PC/DOPE/SA 4:4:2 and PC/DOTAP/Chol 5:2:3). Subsequent studies confirmed that drugs encapsulated within cationic liposomes exhibits greater antibacterial efficacy, probably due to their ability to target bacteria biofilms through electrostatic interactions, thus allowing the drug release close to the pathogen. Messiaen et al. (2013) reached the same results by encapsulating tobramycin in neutral, anionic and cationic liposomes. The greater efficacy of drugs loaded within liposomes was confirmed by Mugabe et al. (2005), who encapsulated the antibiotic gentamicin within liposomes of different composition (DPMC, DPPC, and DSPC). Results showed an increased efficacy of drug activity against PA as compared to free gentamicin.

Joshi and Misra (2001) carried out a study on liposomal (EPC-Chol) budesonide (an anti-inflammatory corticosteroid) in a dry powder formulation. This system was found to keep the desired drug levels in the lungs for a prolonged time, possibly allowing a better drug efficacy and lower adverse effect. Although positively charged liposomes showed better results on the increase of drug activity against several pathogens, they were found to be more toxic for human lung cells than uncharged or negatively charged systems (d'Angelo et al., 2014; Kuzmov and Minko, 2015); this evidence opened to the development of new strategies in order to design new liposomal formulations with less adverse effects on lung cells. Currently, two more antibiotics have been developed as liposomal formulations. The first is amikacin liposome inhalation suspension (Arikayce®, Insmed, Monmouth Junction, NJ, USA), which has completed the phase III of clinical trials and it has been approved by FDA in 2018. It consists of neutral liposomes (DPPC: Chol), with a mean size of 0,3 μm, provided as a sterile aqueous liposomal suspension administrated through an eFlow® Nebulizer. The second is liposomal ciprofloxacin (Pulmaquin® and Lipoquin®, Aradigm Corp., Hayward, CA, USA), which has completed phase II of clinical trials, showing a superior drug efficacy with respect to non-encapsulated ciprofloxacin (Paranjpe and Müller-Goymann, 2014; Moreno-Sastre et al., 2015). Due to the intrinsic structure of liposomes, a further step on the development of new liposomal formulations has been the encapsulation of more than one drug within the bi-layered structure (Van Rijt et al., 2014). Halwani et al. (2008) in 2008 tested the antimicrobial efficacy of a liposomal gentamicin formulation with Gallium metal (Lipo-Ga-GEN). Results indicated an improved efficacy of this multidrug formulation with respect to gentamicin alone in eradicating antibiotic-resistant PA.

As reported previously in this review, cationic liposomes are the most widely investigated systems to deliver DNA intracellularly. Manunta et al. (2017) developed a self-assembling nano-complex constituted of a DOTMA:DOPE liposome, an epithelial targeting peptide and siRNA (small interfering RNA, which represents an attractive approach for the treatment of several pulmonary diseases characterized by an over-expression of genes) against the α-subunit of epithelial sodium channel (ENaC), thus modulating sodium hyperabsorption and helping to restore the mucus hydration and the airway clearance. The authors showed the promising chance to use this approach as co-adjuvant therapy for CF.

Tagalakis et al. (2018) developed siRNA nanocomplexes that could mediate silencing of airway epithelial sodium channel (ENaC) with functional correction of epithelial ion transport. Receptor-targeted nanocomplexes (RTNs) are made of cationic liposomes and targeting peptide mixed with siRNA. In this study it was observed that NPs mediated delivery of siRNA offered an effective delivery by correcting mucociliary defects in human CF cells *in vivo*. A phase IIb clinical trials testing the efficacy of a non-viral gene therapy for CF has been carried out by Alton et al. (2015). In this study, the author complexed the plasmid DNA pGM169 with GL67A liposome and administered the complex to CF patients once a month for 1 year through a nebulizer. The results showed a statistically significant increase in FEV_1_, suggesting lung function stabilization and demonstrating the good potential of the pGM169/GL67A formulation as a safe, non-viral strategy for CF gene therapy (Trial ID: NCT00789867).

The main issues about liposomes as drug delivery for the treatment of pulmonary chronic diseases, such as CF, arise from the need to develop inhalable formulations which have to be delivered through nebulizers (d'Angelo et al., 2014). In fact, the nebulization process may affect liposomes stability and causes their aggregation. For this aim, several strategies are being investigated, such as lyophilisation (Chen et al., 2010), spray drying (Willis et al., 2012) and supercritical fluid technology (Misra et al., 2009), in order to stabilize liposomes and possibly achieve DPI liposomal formulations; some of these have shown promising potentials for pulmonary drug administration, but are still in an early development stage.

### Solid Lipid Nanoparticles

Solid lipid nanoparticles (SLNs) are lipophilic particulates (Yang et al., 2008) which have been studied as drug delivery systems since 1990s. In contrast to liposomes, they don't have a bi-layered structure, but are made of lipids which form a crystalline matrix, solid at room and body temperature, with mean diameters ranging from 40 to 1,000 nm (Weber et al., 2014). The most used solid lipids are fatty acids, triglycerides, steroids, phospholipids and waxes (Zhang et al., 2010; Paranjpe and Müller-Goymann, 2014). The most remarkable advantages of SLN are physical stability and low cytotoxicity, along with the possibility of easy, solvent free scaling-up processes (Mehnert and Mäder, 2012; Weber et al., 2014). However, their main limitation is represented by a low drug loading capacity, which is caused by an increased rigidity of the matrix during the storage (Müller et al., 2000).

The second generation of lipid nanoparticles, called lipid nanocarriers (NLC), consists of a solid lipid matrix made of more than one component, reducing NLC rigidity and allowing a higher drug loading. Even though these systems have been extensively studied for oral (Pinto and Müller, 1999; Müller et al., 2006), dermal (Wissing and Müller, 2001; Müller et al., 2002; Pardeike et al., 2009), parenteral (Joshi and Müller, 2009), and ocular (Araújo et al., 2010) administrations, pulmonary applications are still in early development. Various drugs have been encapsulated within SLNs for the treatment of CF and other pulmonary diseases, such as budesonide (Zhang et al., 2011) and ciprofloxacin (Jain and Banerjee, 2008). Amikacin loaded SLNs were developed by Varshosaz et al. (2010, 2013) and Ghaffari et al. (2011). After optimizing the particles morphology and size, they compared the efficacy of amikacin loaded SLNs with respect to the free drug. The results of these studies showed an increased efficacy of the loaded drug, which could be administered in lower doses (or longer intervals) when encapsulated in SLNs.

In 2018, Robinson et al. (2018) developed lipid-based NPs (LNPs) for packaging and delivery of chemically modified CFTR mRNA (cmCFTR) to restore chloride secretion. They demonstrated that cmRNA delivered by LNPs is effectively translated to a protein product that successfully reach cells membrane. Hence, LNP-cmRNA, whose mechanism of action (i.e., increase of CFTR channel opening on apical cell membrane) and efficacy are comparable with those observed for ivacaftor, represent a promising platform for the correction of CF. Finally, although studies on SLNs and NLCs as pulmonary carriers are in the first stages of research, they have shown good therapeutic potential.

### Dendrimers

Dendrimers are highly ordered, branched macromolecules that possess a tree-like architecture. Three regions can be distinguished in their structure: a core, several dendritic branches (called generations) and functional end groups located in the outer layer of the branches (Menjoge et al., 2010; Zhang et al., 2010; Mehta et al., 2019). This peculiar structure, which can be achieved either with divergent or convergent synthetic approaches, makes dendrimers suitable to a wide range of chemical modifications. Moreover, their highly branched nature provides high surface area to size ratio, which can be used to incorporate different chemical species within the structure in a host-guest approach. The first family of polyamidoamine dendrimers (PAMAM) were synthesized by Tomalia et al. (1985) and PAMAMs are now one of the most studied and used dendrimers. Since then, other types of dendrimers have been synthesized and studied for biomedical applications, such as polyetherhydroxylamine (PEHAM), polypropylenimine (PPI), and polyether dendrimers. Due to their unique physicochemical, biological and mechanical properties, these molecules have raised interest in the field of nanomedicine as possible nanoplatforms for antimicrobial and gene delivery. Drugs conjugated to dendrimers can be administered through different routes, such as oral, injection, ocular, nasal, and pulmonary ones. Compared to other nanocarriers commonly used in nanomedicine, dendrimers show some advantages. In fact, by tuning their structure, for example, it is possible to improve the drug functionality thanks to the multivalency effect. Moreover, from a pharmacological point of view, the high monodispersity of dendrimers allows a better uptake efficacy and bioavailability of drugs encapsulated within the structure, with an easier penetration across biological barriers by transcytosis (Menjoge et al., 2010; Mehta et al., 2019).

The main targets of pulmonary therapies using dendrimers as drug delivery systems are lung cancer, chronic obstructive pulmonary disease (COPD), asthma, tuberculosis. bronchiectasis and pneumonia (Rolland et al., 2009). Dendritic formulations to be administered via inhalation have been developed both as inhalable suspensions and dry powders. In 2017, Agnoletti et al. (2017) developed dendrimer-siRNA nanocomplexes for gene therapy, which is an attractive approach for the treatment of several pulmonary diseases characterized by an over-expression of genes. The nanocomplexes developed showed an efficient cellular uptake and a good gene silencing capability compared to other types of carriers. Brockman et al. (2017) evaluated a strategy to improve the bioavailability and targeted delivery of cysteamine, a FDA approved drug with anti-oxidant, anti-biofilm and mucolytic properties. They developed a PAMAM dendrimer whose terminal groups were modified to obtain a cysteamine-like structure (PAMAM-DEN^CYS^) and demonstrated its efficacy in reducing PA infection and growth, along with its ability to rescue the misfolded F508del-CFTR from aggresome-bodies by inducing its trafficking to the plasma membrane. CFTR modulators were also embedded within dendritic structures, as reported in the work by Faraj et al., who standardized the therapeutic efficacy of the novel dendrimer-based autophagy-inducing agent, cysteamine. In their study, they synthesized and tested a novel nano-molecule consisting of cysteamine-core PAMAM dendrimer (G4 90/10 PAMAM DEN^[CYS]^) and demonstrated its efficacy in the treatment of patients with CF, since the nanostructure preserve cysteamine activity while increasing its bioavailability compared to other means of administration (Faraj et al., 2019). Several strategies have been developed to achieve a more effective cellular uptake of drugs-loaded dendrimers, avoiding their inactivation and elimination by immune response and macrophages activity. Among these, the coating with hydrophilic and biocompatible polymers is the most common strategy. PEG (polyethylene glycol) is the most frequently used polymer to coat and protect a variety of nanocarriers due to its ability to offer stealth properties to them and to drug molecules (Suk et al., 2009, 2011; Kaminskas et al., 2014; Taghavi Pourianazar et al., 2014; Zhong et al., 2016).

### Polymeric Nanoparticles

With the rapid development of nanotechnology in the field of nanomedicine, polymeric NPs have gained much importance, as they represent a major class of nanotherapeutics. Polymeric nanocarriers are widely used as drug delivery systems and administered via different routes, including inhalation (Kuzmov and Minko, 2015). The most relevant features of polymeric systems are their ability to perform a prolonged and controlled drug release, stabilize the drugs encapsulated and promote cellular uptake of pharmaceuticals. Moreover, polymeric NPs show great versatility: their morphological and surface properties, such as size, shape and zeta potential can be easily tuned by choosing different polymers length and kinds of surfactants and solvents for the synthesis. Moreover, their properties can be tailored by adding specific functional groups, drug moieties and target ligands on their surface. Among synthetic polymers, poly-lactic acid (PLA) and poly-lactic-co-glycolic acid (PLGA), both FDA approved, are the most widely used (Cryan, 2005) due to their great biocompatibility and biodegradability. In particular, PLGA offers the possibility to tailor its biodegradation rate by modifying its compositional ratio.

Polymeric NPs have been largely used to avoid side effects and prevent the early degradation of drug molecules, and also to improve the pharmacokinetic of drugs with scarce bioavailability. As an example, curcumin has been found to be a non-toxic, low-affinity sarcoendoplasmic reticulum calcium ATPase (SERCA) pump inhibitor which allows F508del CFTR to escape from the endoplasmic reticulum and reach cell membrane (Egan et al., 2004; Zeitlin, 2004). However, previous studies showed a low efficacy of this compound due to its poor absorption and rapid metabolism. In their research, Cartiera et al. (2010) encapsulated curcumin in PLGA NPs in order to increase its absorption and improve its bioavailability. The *in vivo* tests showed that curcumin loaded NPs enhanced the effects of the compound in CF mice compared to free drug.

Vij et al. (2010) developed PEGylated PLGA NPs encapsulating PS-341, a FDA approved proteasome inhibitor, which has been firstly employed for cancer therapy, even though recent studies have proved the importance of this class of drugs in pharmaco-gene therapy of CF. The authors of the study obtained a controlled and sustained delivery of drug, increasing its efficacy against PA infections.

Ciprofloxacin was successfully encapsulated in PLGA NPs by Günday Türeli et al. (2017), who evaluated the efficacy of the nanocomplex against PA strains, along with its ability to cross the mucus. Embedding the drug within a polymeric matrix allowed a high percentage of loading, while the nanometric size and surface properties permitted a good penetration of the bioactive payload within the mucus. Additionally, an enhanced antimicrobial activity of nano-formulated ciprofloxacin at lower drug doses was reported, indicating its promising potential as drug delivery system for CF antimicrobial treatment. Similar results were reported by Ernst et al. (2018) who encapsulated tobramycin within PEG-coated PLGA nano (≈230 nm) and micro (≈900 nm) particles and observed an enhance antimicrobial activity, along with a good penetration within mucus and bacterial biofilm in dynamic physiological conditions.

As reported above in this section, NPs have been tested as carriers not only for antimicrobial treatments but also for gene therapy. Haque et al. (2018) studied the efficacy of cmRNA^hCFTR^ encapsulated in chitosan-coated PLGA NP's delivered to the lungs of a CFTR deficient mice by intra-venous and intratracheal administration. In this study, they observed a reduced chloride secretion and a general restore of critical lung function parameters, including a noticeable increase in FEV_1_, suggesting that NP-cmRNA^hCFTR^ could be promising therapeutic option for CF patients. Another strategy used to overcome the issues of storing and to improve dispersion and deep lung deposition of NPs consists in the use of NEMs formulations (Muralidharan et al., 2015). In these preparations, NPs can be: (i) encapsulated in polymer-based carriers, (ii) loaded on the polymer surface or (iii) dispersed in a polymer matrix. In all these cases, the polymeric part dissolves upon exposure to the lung environment and releases NPs (Sung et al., 2007).

An attractive new class of non-viral gene delivery vectors is represented by poloxamine-based block copolymers (Richard-Fiardo et al., 2015). Poloxamines are x-shaped block copolymers constituted of poly(ethylen oxide)/poly(propylen oxide) (PEO/PPO) blocks bonded to a central ethylenediamine moiety. Poloxamines are commercially available in a wide range of PEO/PPO ratios and molecular weights under the tradename Tetronic®. Their peculiar structure confers several features, such as temperature and pH sensitiveness and ease of core modification. Due to their hydrophobic core, these nanocarriers are used to solubilize and stabilize poorly water-soluble drugs (Alvarez-Lorenzo et al., 2010). Despite their potential as non-viral vectors for gene therapy, poloxamines alone are not able to overcome some barriers posed by the *in vitro* transfection, while *in vivo* they exhibit a lower efficiency than viral vectors. Guan et al. (2019) developed a platform of synthetic peptides able to self-assemble to poloxamines and nucleic acids to form compacted NPs. The author demonstrated that the use of these NPs led to the enhancement of both mRNA and plasmid DNA expression both *in vitro* and in the lungs of CF mice, without showing any toxicity, thus providing a new strategy for the development of non-viral gene delivery.

## Conclusions and Future Remarks

Nanomedicine represents an extraordinary opportunity for the improvement of current therapies and for the development of innovative treatment options for CF previously considered hard or impossible to treat. The huge amount of research on NPs development as nano-carriers for gene and drug delivery directly to the lungs by inhalation has the opportunity to strongly modify symptomatic treatments for CF patients, as well as those based on CFTR modulators and gene therapies. Due to the peculiar environment in which the therapies have to operate, characterized by several biological barriers (pulmonary tract, mucus, epithelia, bacterial biofilm), the use of nanotechnologies to improve and enhance drug delivery or gene therapies seems to be an extremely promising way to be pursued. However, the road to a definitive realization is still long, although several nano-systems were already tested with successful results with FDA-approved drugs. A special attention should be paid in the use of biocompatible and biodegradable inhalable NPs, that must be well-tolerated *in vivo* as in the case of other disease treatment (Di Mauro et al., 2016; Miragoli et al., 2018). *In vitro*/*in vivo* studies represent a critical step before the selection of the best formulations to candidate for use in humans. Therefore, there is an impelling need of *in vitro*/*in vivo* CF models to acquire knowledge on the safety of the nano-carriers in the lungs. Not only traditional tests, but also newer models should be used to shed light on the possible mechanisms of NPs toxicity. In particular, the shortage of chronic toxicological data on NPs is one of the big gaps to be closed. Once these challenges will be addressed, sustained-release nanocarriers would represent a real benefit for both pharmaceutical companies working to develop novel inhaled products and patients suffering from CF.

The studies on genetic therapies as well as those based on CTFR modulators are already at an advanced stage, but not yet optimal and in some case with results not yet inclusive for a large part of CF patients. Therefore, it should also be considered that symptomatic therapies have not finished their function, indeed they need to be open to new solutions, because we believe that, unfortunately for a long time to come, they will be indispensable for many patients. In this domain, bacterial and fungal infections can be fought by the use of appropriate delivery of antibiotics mediated by NPs while inflammations, due to the peculiar physiological state of the patients, can be circumvented with the same approach by the use of anti-inflammatory drugs.

The genetic defects of CFTR channel may be restored by using highly specific drugs (i.e., modulators) targeting the defective channel protein restore, at least in part CFTR functions. In this latter case, patients treated with these drugs experienced severe systemic side effects and to boost their efficacy the drugs concentration and their permanence at the lungs, the major site of disease, should be improved. The pulmonary administration of CFTR modulators based on mucus penetrating NPs could be an effective strategy, potentially increasing higher local drug concentrations and minimizing side effects when compared to the oral route of administration (Porsio et al., 2018).

Together with these strategies, various different advanced gene therapies seem to be the most promising to eradicate CF from patients in a definitive way, especially if these therapies will be enhanced by the use of nanotechnology in an appropriate way. Overall, genetic editing tools such as CRISPR/Cas9 look more convincing in producing an effective and definitive resolution of CF disease. The major problem to be addressed, is the possibility that Cas9 recognizes sequences different from the target one and therefore introduces unpredicted changes. In fact, the RNA fragment that guides Cas9 admits some incorrect pairing, making Cas9 able to cut even regions of DNA other than the target. This issue can currently be very limited by identifying very selective RNA guide and using alternative or modified Cas9 that allow greater specificity, such as the one developed by Casini et al. (2018). The same authors and collaborators (Maule et al., 2019) developed a genome editing strategy to repair 3272-26A>G and 3849+10kbC>T CFTR mutations. Both mutations alter the correct splicing of the CFTR gene leading to a premature termination codon and consequent expression of a truncated non-functional CFTR protein. In this study, the AsCas12a nuclease (from Acidaminococcus sp. BV3L6) and a single CRISPR RNA were used for gene correction in intestinal organoids and airway epithelial cells derived from CF patients carrying the 3272–26A>G or 3849+10kbC>T mutations. The results obtained shown highly precise genetic repair with a complete absence of detectable off-targets. Even more recently, Ricciardi et al. (2018) applied gene editing in the fetuses of pregnant mice to correct the most common F508del mutation in the CFTR gene. They used donor DNA and synthetic molecules that mimic DNA, called peptide nucleic acids, delivered by biodegradable polymeric NPs to target the CFTR gene. Cell's own DNA repair pathways were activated by the triple helix formed by the NPs linked to the target gene in order to fix the mutation in fetal cells. The lungs of mice born with the repaired mutations, appeared normal without obvious signs of inflammation. The team will continue to study mice organ health. Despite clinical trials are years away, Ricciardi theorized that the therapy in humans would be safely deliver at around 18 weeks of gestation.

We must obviously be extremely careful, considering the enormous quantity of preclinical studies in animal models to be carried out before a possible application in a human environment, but it is inevitable to think that *in utero* DNA editing could constitute the new frontier of medicine and project us far ahead in the treatment of diseases like CF. Today, thanks to amniocentesis and non-invasive prenatal tests performed on blood samples, we can identify serious and dangerous genetic diseases in great advance. Tomorrow, thanks to genetic editing protocols, we hope to be able to defeat them definitely.

## Author Contributions

CV and MI have designed and written the manuscript. AA, FC, FB, AV, DC, and MS have proofread and given comments as well as suggestions. MI has supervised and finalized the manuscript.

### Conflict of Interest

The authors declare that the research was conducted in the absence of any commercial or financial relationships that could be construed as a potential conflict of interest.
